# Supplementary material for: Characterization of the Environmental Plasmidome of the Red Sea
Source: Microbiol Spectr. 2023 Jul 3;11(4):e00400-23. doi: 10.1128/spectrum.00400-23 (PMC10434023; doi:10.1128/spectrum.00400-23)
Supplement: Supplemental file 9 — Fig. S1 and Fig. S2. Download spectrum.00400-23-s0001.pdf, PDF file, 0.2 MB [file spectrum.00400-23-s0001.pdf]

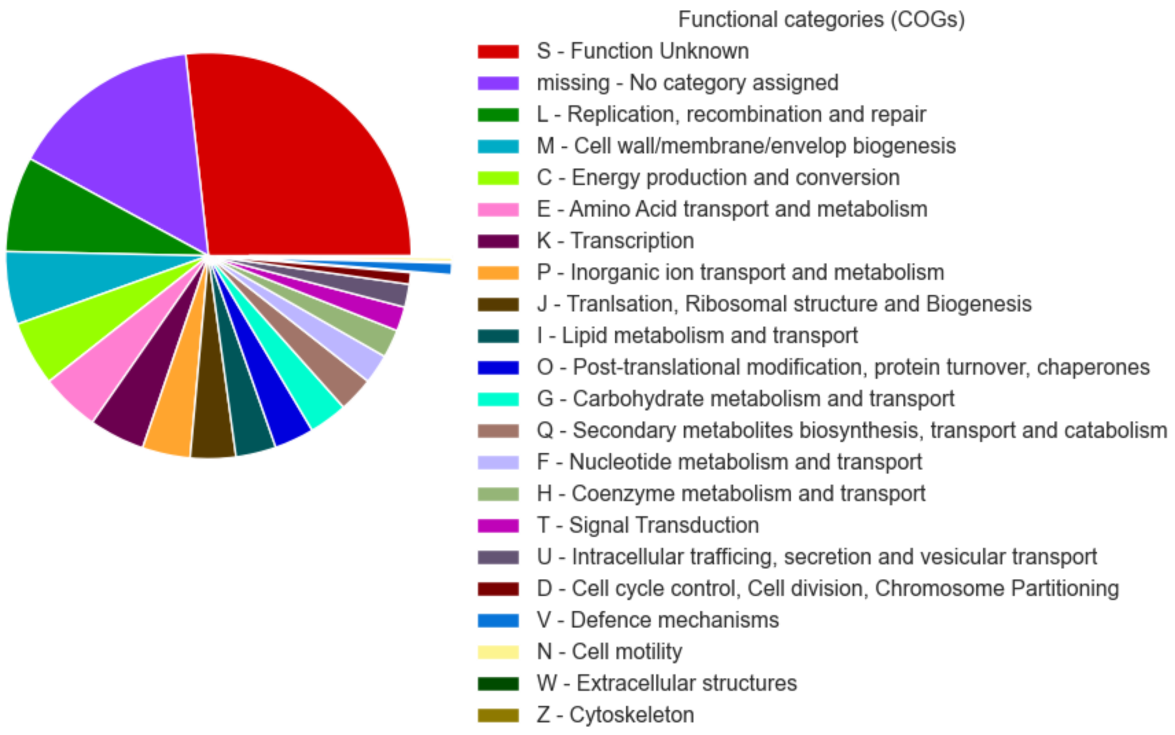

Fig.S1 Functional classification of COG categories in each sampling point using EggNOG. COG category is represented by color, COG category frequencies are represented by size of the pie chart slices. Categories names are shown on the right, in the same order.

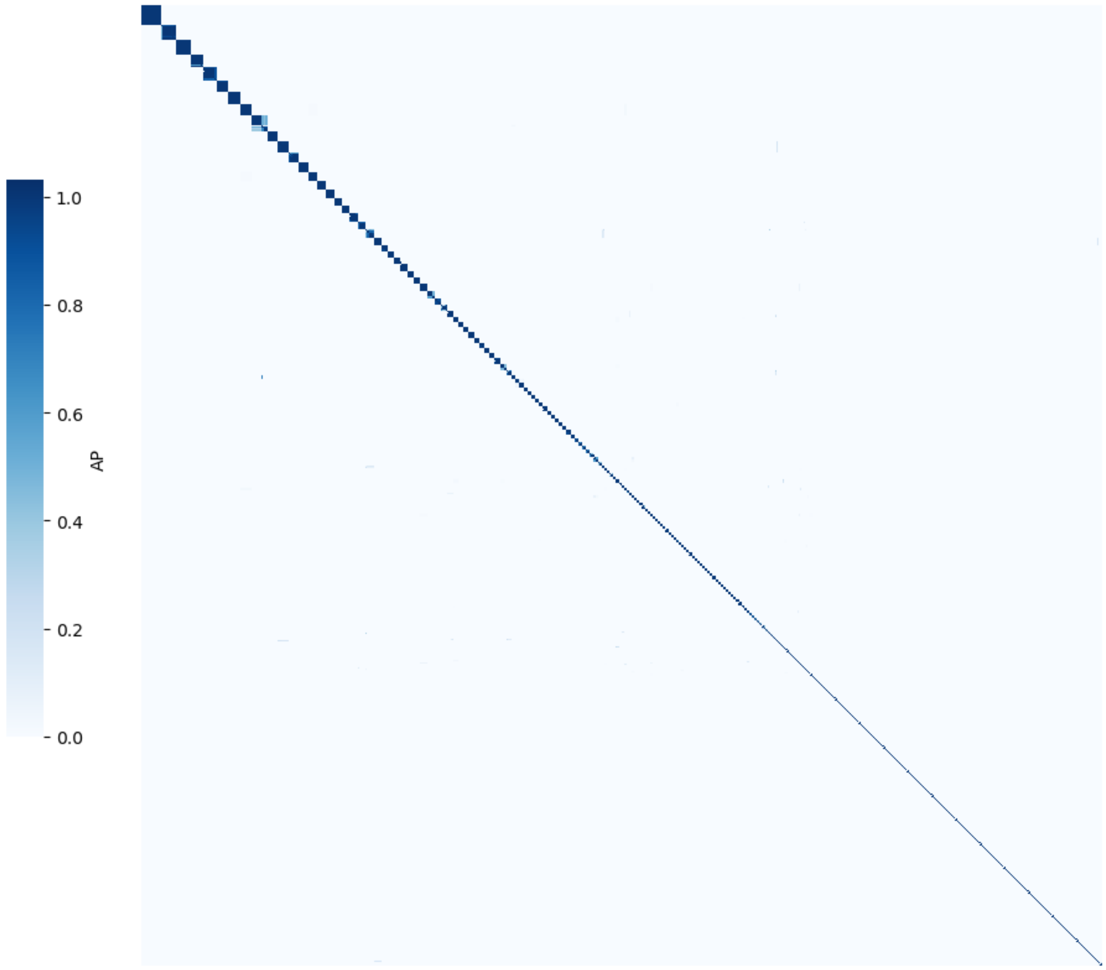

Fig. S2. A heatmap representing the alignment ratio (AP: 1.0 – 100% coverage, 0.0 – no coverage) between all plasmid candidates. Each row and each column represent a plasmid candidate. Alignment percentage for dendrogram clustering was calculated as ratio of alignment length to minimal of the query or subject length, while alignment percentage for heatmap was calculated as ratio of alignment length to query length.
